# Personal

**Published:** 1902-07

**Authors:** 


### PERSONAL.

Dr. Frank Billings, of Chicago, was elected president of the American Medical Association at the recent annual meeting held at Saratoga, N. Y. He has been known among Chicago's medical practitioners and educators nearly twenty years. He was graduated from Chicago Medical College, now the medical department of Northwestern University, and, after a year spent as an interne at the Chicago Hospital, engaged in post-graduate work at Vienna and Paris. Returning to Chicago, he accepted the chair of professor of medicine in Chicago Medical College, resigning to accept a similar position with Rush Medical College. He is now the dean of the latter college, and the head of its medical department. He is also attending physician to the Presbyterian and Cook County hospitals.

Dr. Billings is a man in the vigor of his manhood and is a credit to the American profession. His appearance is indicative of everything that may contribute to a successful administration of the great office to which he has been called.

Dr. Eugene Smith, of Detroit, U. of B., '66, recently paid a visit to Buffalo where he was welcomed by many of his old friends.

---

Dr. James Wright Putnam, of Buffalo, was elected president of the American Neurological Association at its twenty-eighth annual meeting, held in New York, June 5-7, 1902.

---

Dr. and Mrs. Allen A. Jones, of Buffalo, sailed about the middle of June to spend two months in Europe.

---

Dr. John O. Roe, of Rochester, read a paper entitled, Deformities of the nose, and their repair, at the semi-annual meeting of the Medical Society of the County of Erie, June 17, 1902. It was an interesting exposition of ingenious and skilful surgery and was received with marked attention.

---

Dr. Edwin Ricketts, of Cincinnati, President of the American Association of Obstetricians and Gynecologists, has been elected professor of abdominal and gynecological surgery in the Cincinnati College of Medicine and Surgery.

---

Dr. and Mrs. Herman Mynter, of Buffalo, sailed June 17, 1902, on the "Patricia" for Plymouth, Eng. Their two daughters, Emily and Agnes, will proceed with them to Switzerland.

---

Dr. Sydney A. Dunham, of Buffalo, goes abroad about the first of July for a three months' stay.

---

Dr. Reger Cutting, of Buffalo, has been appointed medical officer on the steamship "Northwest." In the fall Dr. Cutting expects to take a position on one of the Pacific steamships as surgeon.

---

Dr. Charles R. Borzilleri, of Buffalo, has been appointed attending physician in the department of diseases of children of the Buffalo Hospital of the Sisters of Charity.

---

Dr. W. E. Parker, of New Orleans, located at Hot Springs, Ark., June 15, 1902, to continue the practice of his profession.

Dr. Alexander McNeil Blair, of Buffalo, sailed on the "Zealand" for Antwerp, June 4, 1902, to be gone until about September 1. Dr. Blair will spend most of his time in the interior of Germany. He goes for study and pleasure.

Dr. John S. McFarland, of Buffalo, was graduated from Johns Hopkins University June 18, 1902, and will soon go to Germany for a special course in medicine and surgery, to be absent a year.

Professor O. Haab, of Zurich, the eminent oculist, visited Buffalo recently the guest of A. A. Hubbell. After a day at Niagara Falls, Dr. Hubbell entertained Professor Haab at luncheon at the Saturn Club together with several Buffalo physicians.

